# To Our Young Men—A Caution

**Published:** 1880-11

**Authors:** 


*To Our Young Men.—A Caution.*—Nothing is more captivating and seductive to a tyro than the witnessing the performance of clever and elegant operations by experts. The novel methods, the dextrous manipulation, the beautiful results are seen, and the endeavor is made to imitate them; often and usually the imitation being a very bad one indeed. More especially are young men apt to imitate the *rapid operations* in dentistry they see at the hands of those giving demonstrations, and this is written as a caution, to them seemingly useless, yet really important. Rapidity with excellence can rarely be attained, and if attained, the two come only from long years of patient practice and training. Often they altogether refuse to go hand in hand, and in that case one must be content to be excellent and not rapid. Most of the really good dentists known to the writer are slow, doing very little work in a day.

We write then this caution to our young men, who are watching in the hope to catch the rapid yet sure strokes of some man of genius whose work they see. Strive first and all the time for the excellence you see, and hope for the dexterity. If it comes, well—if not, do not sacrifice any of your precision and painstaking for it. H.

